# A Survey-Based Study on Physical Activity Promotion for Individuals with a Current or Past Diagnosis of Cancer in Canada

**DOI:** 10.3390/curroncol29120770

**Published:** 2022-12-13

**Authors:** Jenna Smith-Turchyn, Catherine M. Sabiston, Elizabeth Ball, Som D. Mukherjee

**Affiliations:** 1 School of Rehabilitation Science, McMaster University, 1400 Main Street West, IAHS Rm 436, Hamilton, ON L8S 1C7, Canada; 2 Faculty of Kinesiology and Physical Education, University of Toronto, 55 Harbord St., Toronto, ON M5S 2W6, Canada; 3 Faculty of Health Sciences, McMaster University, 1200 Main Street West, Hamilton, ON L8N 3Z5, Canada; 4 Department of Oncology, McMaster University, 1200 Main Street West, Hamilton, ON L8N 3Z5, Canada

**Keywords:** exercise, education, translational science, neoplasms

## Abstract

Purpose: To determine the prevalence and content of discussions regarding physical activity (PA) promotion between individuals with a current or past diagnosis of cancer and their oncology care team. Methods: Design and Procedure: A cross-sectional survey on PA discussion between individuals with a current or past diagnosis of cancer and their oncology care team was conducted at a single timepoint. Participants: Eligible participants were adults with a current or past diagnosis of cancer at any time point in their cancer treatment who had a pre-scheduled appointment with their oncology care team. Results: A total of 100 participants completed the survey. PA-related discussions happened in 41% of the patient-provider interactions and 66% of respondents reported PA discussions at some point during care. No significant association occurred between cancer type, stage, or treatment status and PA discussions at any timepoint (all *p*’s > 0.05). Most respondents were satisfied with the education provided on PA (54%); however, only 37% were sufficiently active. Those receiving education from their medical oncologist were more likely to be ‘sufficiently active’ (*p* = 0.020) according to the Godin Leisure Time Exercise Questionnaire. Conclusions: Most respondents discuss PA with an oncology care provider at some point during their cancer treatment; however, few are sufficiently active. Future research is needed to determine strategies to facilitate PA promotion and close the gap between discussions and actual physical activity behavior.

## 1. Introduction

The last decade has seen a plethora of research supporting the use of regular exercise to manage common side effects of cancer treatment [1]. Benefits of various forms of exercise have been found to manage physical side effects such as fatigue [1,2,3], lymphedema [1,4,5], and reduced muscle and bone health [6], as well as psychosocial effects such as reduced anxiety [1,7] and depressive symptoms [1,8,9]. Together, these beneficial effects lead to improvements in overall functioning and quality of life in individuals living with a current or past diagnosis of cancer [1,10,11]. Additionally, recent evidence has found that regular exercise participation can reduce all cause and cancer-specific mortality in those with breast, prostate, and colon cancer, as well as prevent cancer recurrence [12,13]. These benefits of exercise appear to be related to complex biological and functional changes affecting multiple different organ systems within the body, as well as due to improvements in comorbid conditions such as obesity, cardiovascular disease, and diabetes [12]. Translational research has found that exercise improves markers of inflammation and immunity with decreases in c-reactive protein and improved mobilization of natural killer cells, T-cells, and B-cells [12,14,15,16]. Moreover, improvements in the levels of circulating sex hormones and circulating insulin are evident [14,15,16]. Several studies have found cardiovascular functioning and chemotherapy treatment completion rates were improved following exercise and both of these outcomes have been associated with decreased mortality [12,17,18].

Recently, national organizations have published updated exercise guidelines for individuals living with a current or past diagnosis of cancer [1,19]. Recommendations include moderate intensity aerobic training three times per week for 30 min per session, and twice weekly resistance training for all major muscle groups [1]. Resistance training should be performed at an intensity of at least 60% of a 1 repetition maximum [1]. These recommendations have proven safe for this population at various phases in cancer treatment (pre, during, and post treatment) [1]. Despite the published benefits of exercise and available guidelines, only approximately 30% of individuals living with a current or past diagnosis of cancer were found to be performing physical activity at the level recommended by current guidelines [20,21,22,23].

Much research has described barriers to exercise participation from a patient’s perspective [21,24,25]. Barriers span both individual, institutional, and educational factors. Individual factors include physical challenges, such as pain, fatigue, and other physical side effects; institutional factors include a lack of exercise resources and a lack of funding for exercise programs and professionals; educational factors include an unawareness of exercise benefits and the need to exercise, as well as an unawareness of available exercise programs [21,24,25]. Further, patients describe a lack of access to and engagement with exercise programs and qualified exercise professionals (QEP’s), including physiotherapists or exercise physiologists [26].

From the perspective of the health provider, a survey study published in 2017 found that approximately 80% of oncology care providers at a Canadian institution were not aware of exercise guidelines for this population [27]. While respondents in this study described a desire for further training around exercise and physical activity for individuals living with a current or past diagnosis of cancer, these health professionals reported low current knowledge on when, how, and which patients to refer to an exercise program [27]. Barriers to exercise promotion from the perspective of the oncology care provider include institutional factors (time, lack of role on who should discuss exercise, funding), health professional factors (lack of exercise knowledge, do not exercise themselves), and perceived patient factors (describe patients as having little time, negative attitude toward exercise, side effects that limit participation) [27,28,29]. Nonetheless, individuals with a current or past history of cancer indicate that they are interested in receiving exercise-related advice from their oncology care providers [20], and value recommendation and reinforcement of trusted health professionals throughout cancer treatment [30].

To overcome these barriers, guidelines recommend that oncologists “Assess, Advise, and Refer” individuals living with a current or past diagnosis of cancer to the most appropriate exercise service [1,20]. An effective pathway of referral from an oncology clinician to an exercise program is needed to facilitate increased exercise behavior in this population [31]. As research in exercise oncology continues to move forward quickly, it is important to determine if there has been any improvement in healthcare provider promotion in clinical practice. Therefore, the purpose of this study was to explore the prevalence and content of discussions regarding physical activity promotion between individuals living with a current or past diagnosis of cancer and their oncology care team at a cancer institution in Ontario, Canada.

## 2. Materials and Methods

### 2.1. Study Design

This study used a cross-sectional survey to gather data on physical activity discussion between individuals living with a current or past diagnosis of cancer and their oncology care team at pre-scheduled appointments. The Strengthening the Reporting of Observational Studies in Epidemiology (STROBE) guidelines were used when creating this report [32].

### 2.2. Participants and Setting

Participants were recruited from the Juravinski Cancer Centre (JCC) in Hamilton, Ontario. Eligible participants were adults (>18 years of age) with a current or past diagnosis of cancer (any stage and type of cancer) at any time point in their cancer treatment (during or post treatment) who had a pre-scheduled appointment with their oncology care team at the JCC. The JCC, while located in Hamilton, Ontario, serves a wide geographical range and is a regional referral center for central-west Ontario, Canada [33]. The JCC administers cancer treatments, such as chemotherapy and radiation therapy, and is strictly an outpatient facility (there are no overnight beds) [33]. A wide variety of healthcare providers work in the clinics at the JCC, including medical, radiation and surgical oncologists, palliative care physicians, oncology nurses (specialized oncology, advanced practice, clinical trial, supportive care, and palliative care nurses), social workers, psychologists and registered dieticians [34]. The sample size target for the study was 100 respondents based on suggestions for survey study research using continuous data with alpha set at 0.05 and margin of error at 0.03 [35,36].

### 2.3. Procedure

The Hamilton Integrated Research Ethics Board (HiREB) approved this study (ID#: 10572). Potential participants were invited by a research assistant to complete a survey following a regularly scheduled appointment with their oncology care team. Surveys were completed in the JCC Clinic D waiting room in June and July 2021 and took approximately 10 min to complete. A research assistant explained the study to potential participants and had interested individuals sign a consent form. Respondents then completed the survey in hard copy and returned it to the research assistant prior to leaving the JCC. Respondents were given a $5 gift card for completing the survey. While the study team had a physical presence in the clinic during data collection, the oncology team was not aware of the content/questions being discussed within the survey. Participants were only made aware of the contents of the survey after their visit was complete in order to assess what was discussed naturally in the visit interaction.

### 2.4. Instruments

The survey included 4 parts and 14 questions: (1) questions on demographics and cancer characteristics (i.e., age, sex, cancer type, cancer stage); (2) questions regarding the interaction with oncology care providers (i.e., Did you discuss physical activity with your oncology care provider at today’s appointment? If yes, what did the discussion include?); (3) satisfaction with physical activity education provided (measure on a 7-point Likert scale from 1 (extremely dissatisfied) to 7 (extremely satisfied)); and (4) the Godin Leisure Time Exercise Questionnaire to assess physical activity level. Questions included multiple choice (responders select one response), multi-selection (responders select all responses that apply), scaling questions (7-point Likert scale), and open-ended questions.

The Godin Leisure Time Exercise Questionnaire assesses how often in the last week respondents have participated in strenuous (exercise where heart beats rapidly, such as running or vigorous swimming), moderate (increases heart rate but not exhausting exercise, such as fast walking or easy bicycling), or mild physical activity (minimal effort activity, such as easy walking) for 15 min or more [37]. This scale reliably classifies responders into sufficiently active (a score of ≥24 corresponds to meeting exercise guidelines) or insufficiently active (score of <24 corresponds to not meeting exercise guidelines) categories based on a leisure score index (LSI) [38]. This outcome is extensively used to assess physical activity behavior in cancer populations [38,39] and demonstrates internal consistency values ranging from 0.31 to 0.53 when compared to accelerometer data [38]. See Supplementary Material S1 for a copy of the survey used.

### 2.5. Data Analysis

Data from the hard paper copies of the surveys were transferred manually into a secured Excel spreadsheet (Microsoft^®^ Excel for Mac, Version 16.43, Redmond, WA, USA) by research assistants. Survey questions were analyzed using descriptive statistics (using frequencies, percentages, means, standard deviations where appropriate). Exploratory analyses were performed to determine if associations exist between cancer characteristics (type, stage, treatment timepoint) and physical activity discussion between patient and the oncology care team, respondents’ physical activity level, and satisfaction with physical activity education using Chi-square tests. Open ended survey questions were analyzed using content analysis [40] to determine details of physical activity-related discussions. All quantitative analyses were conducted in STATAv15 with significance set at *p* < 0.05.

## 3. Results

### 3.1. Participant Characteristics

A total of 100 participants completed the survey and recruitment was ceased. Participant characteristics are described in Table 1. Most respondents were females (76%) diagnosed with breast cancer (61%). Fifty percent were diagnosed with grade 0-III cancer and 25% had metastatic disease. Most respondents were currently on treatment (64%).

### 3.2. Physical Activity Discussion with Oncology Care Team

Physical activity-related discussions occurred in 41% of the patient-provider interactions. Most of these discussions happened between the patient and medical oncologist (23%) and included an assessment of current physical activity levels (34%), a description of the benefits of exercise for cancer populations (25%), and a discussion on types of exercise to perform (21%). Table 2 provides further detail on physical activity discussion between respondents and their oncology care team. Open-ended survey questions revealed that, in some instances, the oncology care team also gave general advice on how to stay active in daily life to benefit physical health (6 of 28 responses), how to manage physical side effects such as pain, fatigue, and paresthesia when taking part in exercise (4 responses), how to recognize when rest is needed (3 responses), and how to overcome depression and low motivation when trying to exercise (2 responses).

For those who did not discuss physical activity with their provider at the visit (n = 58), 43% (n = 25) stated that they had discussed physical activity with their oncology care team in the past. Overall, 66% of respondents had physical activity-related discussions with their care team at some point during care. Only 24% of those who did not discuss physical activity at the current appointment stated that they would have liked to discuss the topic. In open-ended questions some of these responders said they wanted more information on specific exercise parameters (type of exercise, intensity, number of sessions; 7 of 58 responses), how to be active with current limitations (2 responses), available exercise programs (1 response), and impacts of exercise on physical health and recurrence rates (1 response).

Further analysis demonstrated no significant association between cancer type, cancer stage, or treatment status and physical activity-related discussion at the current visit or at any timepoint (all *p*’s > 0.05). See Table 3.

### 3.3. Satisfaction with Physical Activity Education

The median satisfaction score was 6/7 (moderately satisfied; range 1–7). Most respondents said they were satisfied with the education provided on physical activity throughout their treatment (54% answered a 5, 6, or 7 (satisfied to extremely satisfied) on the 7-point Likert scale), 23% were neutral on the topic, and only 7% said they were dissatisfied with the education provided. See Table 2.

Exploratory analysis found no significant association between cancer type, cancer stage, treatment status, type of oncology care provider who discussed exercise, or number of oncology care providers who discussed exercise and satisfaction with physical activity-related education (all *p*’s < 0.05). However, a significantly greater proportion of those who discussed physical activity at the current visit, or at a previous visit, said they were satisfied with the physical activity education provided throughout their cancer treatment compared to those who had no discussion on the topic (*p* < 0.01). See Table 3.

### 3.4. Physical Activity Level

Only 37% of respondents were considered sufficiently active based on their Godin Leisure Time Exercise Questionnaire LSI scores (see Table 1). Cancer type, cancer stage, treatment status, number of oncology care providers, and satisfaction with physical activity education throughout treatment were not significantly correlated with current physical activity level (*p*’s >0.05). However, of the respondents who discussed exercise with an oncology care provider, the type of provider who discussed physical activity was associated with activity levels. Those receiving exercise education from their medical oncologist (n = 31) were significantly more likely to be ‘sufficiently active’ compared to those (n = 11) who received it from a different type of provider (*p* = 0.02). See Table 3.

## 4. Discussion

This survey-based study examined the prevalence (% of patient/provider interactions that included a discussion of physical activity at one timepoint) and content of discussions regarding physical activity promotion between individuals with a current or past diagnosis of cancer and their oncology care team at a single cancer institution in Canada. Findings revealed that the majority (66%) of respondents had physical activity-related discussions with their oncology care team at some point during care. Most respondents were also satisfied with the education provided on physical activity through their treatment, with those having discussions with their team on this topic being found to rate their level of satisfaction highly. While physical activity promotion was higher than expected, only 37% of respondents were considered ‘sufficiently active’. Those having physical activity-related discussions with their medical oncologists were more likely to be sufficiently active, compared to those who discussed this with other types of oncology care providers. Compared to studies conducted at the same institution 5-10 years ago [21,27], the prevalence of discussions demonstrated in this study was significantly higher (66% now compared to 17% previously [21]). This rate is also promising given a recent systematic review reporting that healthcare professionals self-reported discussing physical activity with cancer patients on average 58.1% of the time [41]. Overall, this change coincides with a rapidly increasing body of literature related to exercise oncology over the past ten years, as well as calls for knowledge mobilization and educational initiatives by those involved in cancer rehabilitation [42,43]. Furthermore, it may be reflective of greater efforts aimed at addressing common barriers (e.g., time pressures, a lack of rehabilitation professionals, limited knowledge of exercise guidelines, and limited referral pathways) [28,29,41]. Further research focused on the oncology care team is needed to understand the potential positive shift in physical activity discussions.

While physical activity discussions occurred at a higher rate than prior studies, the number of respondents who were ‘sufficiently active’ or meeting exercise guidelines for cancer populations was consistent with past reports (~30% [20,22,44,45]). Therefore, an evident gap still exists between physical activity promotion and individual behavior. Upon review of the content of discussions, participants reported that most of the conversations were general with few specific recommendations regarding the actual level of physical activity, and tended not to mention any benefits of exercise on overall function and quality of life. Actionable information (e.g., ‘how’ to do it, where to access exercise-related services), including information on parameters (type, time, duration), was discussed in less than a quarter of the interactions and may reflect the low rate of exercise participation. A recent Delphi survey study including over 300 global stakeholders suggested that in order to implement exercise as a standard of care for individuals with a current or past diagnosis of cancer, top priorities include messaging strategies to support exercise engagement, exercise oncology education models for oncologists and primary care teams, standardized exercise oncology training for diverse exercise professionals across various training environments, qualified exercise professionals’ (i.e., physiotherapist, exercise physiologist) integration into primary cancer care teams, and referral mechanisms to clinical and community based cancer exercise programs [46]. The data from this project support the need for these mechanisms, and tailored exercise pathways, to close the gap between health promotion and behavior [31].

Interestingly, no difference was found in this study in the prevalence of physical activity discussions by cancer type or cancer stage. Furthermore, we did not find any significant differences between physical activity levels and satisfaction with physical activity education between those with different types or stages of cancer. The literature on the benefits of exercise to date has largely focused on early stage (stage I–III) breast cancer [1,47,48,49]; however, benefits have also been demonstrated for many other cancer types [1]. Additionally, while a more cautious and supervised approach to physical activity is recommended for those diagnosed with higher stage cancer (e.g., stage IV; distant metastases), current evidence and guidelines support the use of exercise to ultimately improve overall physical function in this population [50,51,52,53]. A recently published expert consensus on exercising with bone metastases stated that with proper assessment and supervision by a QEP, exercise prescription can follow the general guidelines for cancer populations with an emphasis on postural alignment, controlled movement, proper technique and consideration of bone lesion location and presentation [50].

In exploratory sub-analysis, respondents discussing exercise with a medical oncologist were significantly more likely to be considered ‘sufficiently active’ according to the Godin Leisure Time Exercise Questionnaire. This builds on past literature [54] and highlights the importance that individuals with cancer place on their oncologists’ recommendations [55]. These findings highlight the need for researchers and QEPs to develop standardized strategies that allow medical oncologists to convey physical activity information in an easy and effective way which is manageable in conjunction with the plethora of other information to discuss during clinic visits. In relation to the Exercise is Medicine initiative [56] and suggestion that oncology clinicians “assess, advise, and refer” at regular timepoints during an individual’s treatment [20], it appeared that oncology care providers were assessing levels of physical activity and advising on the benefits, but there was no evidence of referrals. Future research should assess the effectiveness of various promotion and referral approaches, which could include exercise prompts or prescription pads with a list of qualified exercise professionals for referral. Additionally, future research into the optimal time(s) to discuss physical activity, and the number, length, and detail of discussions will help to facilitate promotion strategies for this population in the future.

### Study Limitations

The results of this study should be reviewed with an understanding of its limitations. Firstly, individuals who agreed to complete the survey may have been interested in this topic, which may have introduced bias to the results. Further, this survey was administered at only one cancer center in Ontario, Canada, and most respondents were individuals diagnosed with breast cancer. This may limit the generalizability of results to other institutions or other cancer types. Additionally, this study was conducted in the summer months of 2021, during the COVID-19 pandemic. The pandemic and its associated restrictions may have affected the type and amount of information discussed between the oncology care team and their patients. Lastly, the survey was only administered in English, which may have excluded possible respondents who could not read English.

## 5. Conclusions

In conclusion, this study demonstrated that most individuals with cancer discuss physical activity with an oncology care provider at some point during their cancer treatment. Moreover, most respondents were satisfied with the education provided around physical activity during treatment. However, few were considered sufficiently active. Exploratory sub-analysis found no differences between physical activity discussion, satisfaction, or physical activity level by cancer type or stage of cancer. Those discussing physical activity with their medical oncologist were more likely to be sufficiently active. Practically, these findings highlight the need to determine optimal strategies to facilitate physical activity promotion and to close the gap between physical activity discussions and actual physical activity behavior. This may include the provision of various strategies in clinic, such as using exercise prompts, referral pathways and triaging strategies, or further educational opportunities on physical activity for oncology care providers in order to facilitate accessible ways to engage this population in physical activity during and after cancer treatment.

## Figures and Tables

**Table 1:** Participant characteristics (N = 100).

Characteristics	n & %
Age (years): mean, SD	61 (13)
Sex	
Female	76
Male	23
Did not specify	1
Cancer Type	
Breast	61
Melanoma	9
Prostate	5
Pancreatic	3
Colon	3
Esophageal	3
Squamous cell carcinoma	2
Liver	2
Rectal	2
Ovarian	1
Bone	1
Neuroendocrine	1
Goblet cell carcinoma	1
Soft tissue carcinoma	1
Thyroid	1
Did not answer	4
Cancer Stage	
0	1
1	14
2	22
3	12
4	25
Did not answer	25
Type of appointment	
First appointment	18
Follow up appointment	82
Treatment status	
On treatment	64
Completed treatment	35
Did not answer	1
Treatment type (total n = 64) *:	
Chemotherapy	28
Radiation Therapy	8
Hormone Therapy	19
Other	21
Current activity level	
Sufficiently active	37
Not sufficiently active	62
Did not answer	1

* Note: total number may be higher than 64 due to individuals receiving more than one treatment type.

**Table 2:** Description of physical activity-related discussion with oncology care team (total N = 100).

Content	n	%
Did physical activity discussion happen at today’s appointment?		
Yes	41	41
No	59	59
Who discussed physical activity?		
Medical oncologist	23	23
Radiation oncologist	3	3
Oncology nurse	7	7
Medical oncologist and oncology nurse	7	7
Medical oncologist and other	2	2
Other	3	3
Content discussed		
Current activity level	34	34
Benefits of exercise for cancer populations	25	25
What type of exercise to do	21	21
How to become more active	16	16
How often to exercise or how long to exercise for	14	14
If physical activity was not discussed today (n = 58), has physical activity been discussed in the past with the oncology care team?		
Yes	25	43
No	26	45
If physical activity was not discussed today (n = 58), would you have liked to discuss this topic?		
Yes	14	24
No	33	57
Satisfaction with education provided on physical activity through cancer treatment		
Extremely dissatisfied	3	3
Moderately dissatisfied	1	1
Slightly dissatisfied	4	4
Neutral	23	23
Slightly satisfied	7	7
Moderately satisfied	18	18
Extremely satisfied	29	29
M (SD)	5.35 (1.62)	

**Table 3:** Sub-group analysis of physical activity discussion, activity level, and satisfaction (* bolded values represent a significant difference).

Sub-Group	Discussed Physical Activity at this Appointment (n (%))	*p*-Value	Discussed Physical Activity Now or in Past (n (%))	*p*-Value	Sufficiently Active (n (%))	*p*-Value	Satisfied with Education (n (%))	*p*-Value
Cancer Type								
Breast	21 (34)	0.095	38 (62)	0.478	24 (39)	0.608	31 (61)	0.520
Other	20 (51)		27 (69)		13 (34)		23 (68)	
Cancer Stage								
Early stage (Stage 1−3)	19 (39)	0.447	32 (65)	0.347	18 (37)	0.455	25 (63)	0.355
Advanced disease (Stage 4)	12 (48)		19 (76)		11 (46)		17 (74)	
Treatment status								
Undergoing treatment	25 (39)	0.521	45 (70)	0.187	21 (33)	0.349	39 (66)	0.594
Completed treatment	16 (46)		20 (57)		15 (43)		15 (60)	
Type of OCP ^#^ discussing exercise								
Medical Oncologist	--	--	--	--	15 (48)	**0.021**	26 (87)	0.170
Other	--		--		1 (9)		6 (67)	
Number of OCP’s discussing exercise								
1	--	--	--	--	13 (48)	0.072	21 (81)	0.768
2 or more	--		--		3 (20)		11 (85)	
Satisfaction with physical activity education								
Satisfied	32 (59)	<0.001 *	48 (89)	**<0.001**	20 (37)	0.306	--	--
Dissatisfied	6 (19)		12 (39)		15 (48)		--	

^#^ OCP: oncology care provider; * bolded values: significant difference between groups.

## Data Availability

The data that support the findings of this study are available on request from the corresponding author (JST).

## Supplementary Materials

The following supporting information can be downloaded at: https://www.mdpi.com/article/10.3390/curroncol29120770/s1, Supplementary Material S1: Exercise Prompt—Physical Activity Survey 1.
