# Supplementary material for: A Survey-Based Study on Physical Activity Promotion for Individuals with a Current or Past Diagnosis of Cancer in Canada
Source: Curr Oncol. 2022 Dec 13;29(12):9801–12. doi: 10.3390/curroncol29120770 (PMC9776606; doi:10.3390/curroncol29120770)
Supplement: Supplementary file 1 [file curroncol-29-00770-s001.zip › curroncol-2035808-supplementary.pdf]

Exercise Prompt / Physical Activity Survey - 1

Date: ____________________

Please answer these questions about yourself:

1. What is your month and year of birth? ____
2. Are you male or female? ____
3. What type of cancer have you been diagnosed with? ______________________________
4. What stage of cancer have you been diagnosed with? _____________________________
5. Was this your first appointment or a follow up appointment?

   - ☐ First appointment
   - ☐ Follow-up appointment

6. Are you currently undergoing treatment? ☐ Yes ☐ No

   - ☐ **If YES**, please describe the type of treatment you are currently receiving (check all that apply):
     - ☐ **Chemotherapy**
     - ☐ **Radiation**
     - ☐ **Hormonal Therapy**
     - ☐ **Other (please describe):** ____

       ____

       ____

Please answer the following questions regarding your interaction with your oncology care provider <u>today</u>.

7. Did you discuss physical activity or exercise with your oncology care provider at today's appointment?:

☐ Yes

☐ No

8. If **<u>YES</u>** to question #7, who was this discussion with? (check all that apply)

☐ Medical Oncologist

☐ Radiation Oncologist

☐ Oncology Nurse

☐ Other (please list): ______________________

9. If **YES** to question #7, what did the discussion include?

• Did they discuss your current exercise level?	☐ Yes	☐ No
• Did they discuss the benefits of exercise for cancer survivors?	☐ Yes	☐ No
• Did they discuss how you can become more active?	☐ Yes	☐ No
• Did they discuss what types of exercise you can do?	☐ Yes	☐ No
• Did they discuss how often you should exercise or how long you should exercise for?	☐ Yes	☐ No

What else did you discuss (**related to exercise or physical activity**)?

____

____

____

____

10. If **NO** to question #7, have you discussed physical activity or exercise with your oncology care provider in the past?

☐ Yes
☐ No
☐ Not applicable

11. If **NO** to question #7, would you have liked to discuss this topic (exercise / physical activity) with your health care team today?

☐ Yes
☐ No

12. If **YES** to question #11, what would you have liked to discuss?

____

____

____

____

13. Please rank your satisfaction with the education you have been provided on physical activity / exercise throughout your cancer treatment (please circle):

Extremely Dissatisfied	Moderately Dissatisfied	Slightly Dissatisfied	Neutral	Slightly Satisfied	Moderately Satisfied	Extremely Satisfied

Please answer the following questions about your physical activity level:

During a typical week, how many times (on average) do you participate in 15-minutes or more of strenuous exercise (heart beats rapidly). *Examples include running, jogging, hockey, football, soccer, squash, basketball, cross country skiing, vigorous swimming, long distance bicycling.*

_____ Times

During a typical week, how many times (on average) do you participate in 15-minutes or more of moderate exercise (not exhausting exercise). *Examples include fast walking, baseball, tennis, easy bicycling, volleyball, badminton, easy swimming, alpine skiing, dancing.*

_____ Times

During a typical week, how many times (on average) do you participate in 15-minutes or more of mild exercise (minimal effort). *Examples include yoga, bowling, golf, easy walking.*

_____ Times

During a typical week, in your leisure time, how often do you engage in any regular activity long enough to work up a sweat (heart beats rapidly)?

- ☐ Often
- ☐ Sometimes
- ☐ Never

Thank you for completing this survey!
